# The Social Power of Regret: The Effect of Social Appraisal and Anticipated Emotions on Fair and Unfair Allocations in Resource Dilemmas

**DOI:** 10.1037/xge0000036

**Published:** 2014-11-10

**Authors:** Job van der Schalk, Toon Kuppens, Martin Bruder, Antony S. R. Manstead

**Affiliations:** 1 School of Psychology, Cardiff University; 2 Faculty of Behavioral and Social Sciences, University of Groningen; 3 Department of Psychology/Zukunftskolleg, University of Konstanz; 4 School of Psychology, Cardiff University

**Keywords:** social appraisal, anticipated emotions, fairness, resource allocation, decision-making

## Abstract

We investigated how another person’s emotions about resource allocation decisions influence observers’ resource allocations by influencing the emotions that observers anticipate feeling if they were to act in the same way. Participants were exposed to an exemplar who made a fair or unfair division in an economic game and expressed pride or regret about this decision. Participants then made their own resource allocation decisions. Exemplar regret about acting fairly decreased the incidence of fair behavior (Studies 1A and 1B). Likewise, exemplar regret about acting unfairly increased the incidence of fair behavior (Study 2). The effect of others’ emotions on observers’ behavior was mediated by the observers’ anticipated emotions. We discuss our findings in light of the view that social appraisal and anticipated emotions are important tools for social learning and may contribute to the formation and maintenance of social norms about greed and fairness.

The point is, ladies and gentleman, that greed, for lack of a better word, is good. Greed is right, greed works. Greed clarifies, cuts through, and captures the essence of the evolutionary spirit. Greed, in all of its forms; greed for life, for money, for love, knowledge has marked the upward surge of mankind.

Gordon Gekko, in *Wall Street* (Director: Oliver Stone, 1987)

It was wrong . . . it was greed, pure and simple. I can’t say how sorry I am and how deeply I regret my actions.

Dennis Kozlowski, former CEO of Tyco, at his parole hearing in 2013 after his conviction in 2005 for stealing $134 million from the company.

In everyday life, we are frequently confronted with the need to make a decision about how to allocate resources (e.g., money or time) between ourselves and one or more others. We know that when making such decisions, people consider how they are likely to feel about the consequences of their allocations and make use of these anticipated feelings to guide the choices they make (see Haselhuhn & Mellers, 2005; Mellers & McGraw, 2001; Van der Schalk, Bruder, & Manstead, 2012). The current research examines whether these anticipated emotions and subsequent allocation decisions are influenced by other people’s emotional expressions. Observing someone (like Gordon Gekko in the above quote) expressing pride about being greedy might lead us to anticipate that we would feel good if we were to act in the same way and thereby promote selfish allocation decisions. By contrast, witnessing someone like Dennis Kozlowski expressing regret about his unfair behavior should lead us to anticipate feeling bad if we were to be greedy and promote fair allocations.

Prior research shows that behavior in experimental games is subject to social influence. Bicchieri and Xiao (2009) provided allocators in a dictator game with information about the proportion of subjects in a prior session who had been “fair” versus “selfish”; participants made fairer decisions when they thought prior allocators had been fair (see also Fowler & Christakis, 2010; Krupka & Weber, 2009). In the present research, our main theoretical focus is on how others’ expressions of emotion (rather than their behavior) influence the fairness of allocation decisions.

According to one theoretical perspective, a key way in which emotions regulate behavior is through the anticipation of how we would feel if we were to act in a certain way (Baumeister, Vohs, DeWall, & Zhang, 2007). On this account, the experience of, say, guilt arising from the realization that one has harmed another person shapes future behavior by leaving an “affective residue” that is activated when similar circumstances arise in the future. The anticipation of the negative affect that would be experienced if one were to act in a similar way shapes behavior in the new setting by steering one away from this course of action.

These processes of anticipating affect on the basis of past behavior that had affective consequences are intrapersonal. In the current research, we examine whether interpersonal processes can be integrated into this theoretical account. Social appraisal theory (Manstead & Fischer, 2001) proposes that when we perceive others’ emotional reactions to an event, we infer the underlying appraisals (Hareli & Hess, 2010; Scherer & Grandjean, 2008). This influences social judgments (Mumenthaler & Sander, 2012) and inferences about others’ intentions (de Melo, Carnevale, Read, & Gratch, 2014), and it provides a basis for a shared understanding of the emotional situation and for emotional convergence (Bruder, Dosmukhambetova, Nerb, & Manstead, 2012; Bruder, Fischer, & Manstead, 2014; Parkinson & Simons, 2009). In other words, being exposed to how another person reacts emotionally to an event can influence the way we appraise that event and thereby our emotional responses to it. We propose that this process of social appraisal similarly influences the anticipation of affect. Applied to the issue of resource allocation, this suggests that how others react emotionally to a division of resources that they have made should influence the emotional reaction that we think we would experience when making such a division ourselves.

The Emotion as Social Information model (Van Kleef, 2009) provides another theoretical explanation for the effects of emotion expression on the behavior of observers. According to the model, emotions can influence observers’ behavior by affecting inferences about expressers’ intentions and/or by directly shaping affective reactions. We propose a different route through which emotion expressions can have an effect on the behavior of observers through anticipated emotions.

Previous research on the role of emotions in resource allocation decisions has tended to focus on the role of anger experienced by those on the receiving end of unfair allocations. In the ultimatum game (UG; Güth, Schmittberger, & Schwarze, 1982), the “allocator” decides how to divide a sum of money between self and other by making an offer to the “responder.” The latter can either accept the offer, in which case both players receive the division that was proposed, or reject it, in which case neither player receives anything. According to rational choice models of economic decision-making, the responder should accept any offer, however low. It is well established that offers of less than 20% of the total sum are nevertheless more likely to be rejected than accepted (Bolton & Zwick, 1995; Güth et al., 1982), and this supposedly irrational rejection of unfair offers is related to responders’ anger (Pillutla & Murnighan, 1996; see also van’t Wout, Kahn, Sanfey, & Aleman, 2006; Sanfey, Rilling, Aronson, Mystrom, & Cohen, 2003). The fact that responders’ anger is related to rejection of offers is probably the reason why allocators make more generous offers to responders who shows signs of anger, in order to avoid rejection of their offer (Van Dijk, van Kleef, Steinel, & van Beest, 2008; see also van Kleef, de Dreu, & Manstead, 2004a, 2004b, 2010).

In the van Dijk et al. (2008) study, participants responded to anger expressions of someone with whom they had an interdependent relationship (i.e., their opponent). The effect would likely be different if the emotion came from someone with whom the participant had an independent relationship (e.g., someone with the same role as the allocator). In the latter case, the expression of emotion does not carry information about what the opponent is likely to do, but about the allocator’s own possible emotional reaction to the offer s/he makes. In effect, the other person serves as an exemplar of how one is likely to feel, and such anticipated emotions might influence behavior.

These ideas are examined in the current studies. We test whether learning that another person (exemplar) who felt proud or regretful about allocating resources fairly or unfairly (a) influences a participant’s own allocation behavior when playing the game and (b) does so by influencing the emotions that the participant anticipates experiencing if he or she were to act the same way as the exemplar. We predicted that an exemplar who expresses regret about having allocated resources fairly would elicit less generous offers than an exemplar who expresses pride about having allocated resources fairly; likewise, we predicted that an exemplar who expressed regret about having allocated resources unfairly would elicit more generous offers than an exemplar who expressed pride about having allocated resources unfairly. These effects of an exemplar’s emotion should be mediated by the participant’s own anticipated emotions.

## Studies 1A and 1B

### Method

#### Participants and design

Studies 1A and 1B both had a 2 (behavior: fair, unfair) × 3 (emotion: pride, regret, control) between-subject design. There were 218 participants in Study 1A (*M*_*age*_ = 44.57 years; 61.9% female [2.3% undisclosed]). There were 207 participants in Study 1B (*M*_*age*_ = 46.64 years; 50.7% female). They were recruited through an online loyalty program and received points that could be used for online shopping as compensation. A pilot study using a similar design is reported as supplemental material (S1).

#### Materials

Participants played the UG online and the number of “tokens” or monetary units (MUs) that allocators were willing to share served as a measure of fair behavior. The resource for which participants played was £100, represented by 50 MUs of £2 each. We explained that we would randomly select two pairs of participants who would be paid in accordance with how they played the game. All participants were allocators. After making their offer in the UG, participants were asked to report the minimum MU they would accept as an offer if they were a receiver in the UG. Answers to this question were used to determine acceptance or rejection of offers made by the randomly selected participants, and the £100 resource was divided in accordance with the responses made by participants.

Participants were told that we were interested in their thoughts and feelings about the game. To manipulate exemplar behavior and emotion, participants in Study 1A were given the opportunity to read “a transcript of the thoughts of a previous participant in this experiment.” This person wrote that s/he had considered dividing the 50 MUs in different ways: 45(self)–5(other), 25–25, or something in between these two options. The exemplar behavior manipulation was the choice the exemplar reported having made, which was either a 25–25 split (fair behavior condition) or a 45–5 split (unfair behavior condition). It was also stated that this offer was accepted. To manipulate emotion, the allocator expressed feeling “good,” “proud,” and “pleased” about the decision (pride emotion condition) or feeling “bad,” “sorry,” and “regret” about the decision (regret emotion condition). In the control emotion condition, there was no mention of emotions in the thought transcript. The exemplar behavior and emotion manipulations were crossed to create six different thought transcripts. In Study 1B, we showed participants filmed recordings of exemplars who enacted the thought protocols used in Study 1A. The exact wording of the manipulations and the video recordings are available in the supplemental online material (S2 and S3).

Participants reported their anticipated emotions directly before playing the UG. Depending on behavior condition, we asked: “If you were to divide the tokens in a similar way as the allocator did in the thought transcript you just read [video you just saw] (that is, if you made a 25–25 [45–5] split), to what extent would you feel . . .”. We asked participants to report their anticipated emotions on a scale from 1 (*not at all*) to 5 (*extremely*) for 10 different emotion terms: *pleased*, *proud*, *regretful*, *sorry*, *satisfied*, *relieved*, *embarrassed*, *foolish*, *guilty*, and *ashamed*. We combined three anticipated emotion items into a single anticipated pride scale (*pleased*, *proud*, and *satisfied*; α = .91) and two anticipated emotion items into a single anticipated regret scale (*regretful* and *sorry*; α = .87).

#### Procedure

First, participants provided demographic information and completed a measure of Social Value Orientation (SVO; Van Lange, De Bruin, Otten, & Joireman, 1997). Participants then received instructions for the UG. They were ostensibly randomly assigned to the allocator role. In Study 1A, participants then read the thought transcript that contained the behavior and emotion manipulation, and they completed a set of comprehension checks with feedback to ensure that they understood the rules of the UG. In Study 1B, participants completed the comprehension checks and viewed the film of the exemplar, who was always the same sex as the participant. Participants in both studies then completed manipulation checks, reported their anticipated emotions, and made their UG offer. In the final part of the experiment, participants responded to an open-ended question about their thoughts and feelings concerning the game, and they indicated the minimum number of MUs they would accept as an offer if they were a receiver in the UG. Participants also completed a second manipulation check and a different measure of SVO (Murphy, Ackermann, & Handgraaf, 2011). In Study 1B, we also measured participants’ perceived closeness to the person in the video (Aron, Aron, & Smollan, 1992).

### Results

#### Participants

In Study 1B, 2 participants reported technical problems with the playback of the video, and a further 19 participants did not spend enough time on the video webpage to have seen the entire video. In addition, the comments of one participant in Study 1B showed that this person had not understood the UG. All of these participants were excluded, and 185 participants remained for analyses in Study 1B. No participants were excluded in Study 1A.

Although the participants in Study 1A and Study 1B were recruited on different occasions, everything apart from the medium of delivery of the thought protocol was identical in the two studies. Preliminary analyses of our key dependent measure revealed that study (1A vs. 1B) did not interact significantly with exemplar behavior, exemplar emotion, or their interaction. Therefore, we report the analyses of the pooled data (403 participants in total).

#### Data treatment

Exploration of the data revealed that the key dependent variable was not normally distributed (Kolmogorov–Smirnov: *D*(403) = .22, *p* < .001), and that treating the offer as a continuous dependent variable led to non-normally distributed residuals (the distribution of offers is reported as a histogram in supplemental material S4). Therefore, we decided to dichotomize the main dependent variable: All offers of ≥25 were recoded as 1 = *fair offers* (42.9%) and all offers of ≤24 were recoded as 0 = *unfair offers* (57.1%). We analyzed the effects of our manipulations on the dichotomized offer variable using logistic regression, controlling for study, gender, and all interactions with gender. Analyses of the manipulation checks showed that manipulations were successful. These are reported in the supplemental materials (S5).

#### Offer level

**Figure 1 fig1:**
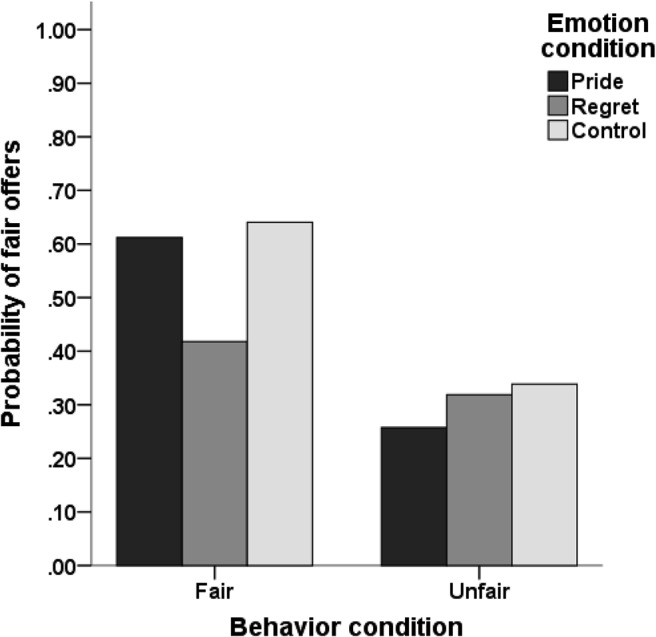
Predicted probabilities of fair offers as a function of exemplar behavior and exemplar emotion (data pooled from Studies 1A and 1B).

**Figure 2 fig2:**
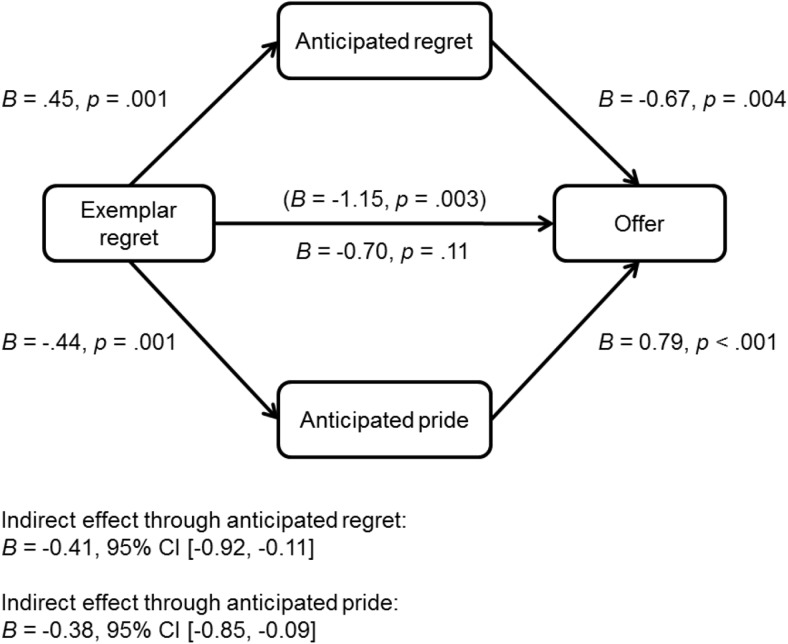
Indirect effect of exemplar regret (compared to control) on odds of making a fair offer through self-reported anticipated regret and pride in the fair behavior condition (data from Studies 1A and 1B).

The expected interaction between exemplar behavior and exemplar emotion was marginally significant, *p* = .052. Simple slope analyses revealed a significant effect of exemplar emotion in the fair behavior condition, *p* = .007, but no effect of exemplar emotion in the unfair behavior condition, *p* = .42. In the fair behavior condition, participants in the regret condition were less likely to make fair offers than were those in the control condition, *B* = −1.15, *p* = .003, odds ratio = 0.32, but there was no difference between the pride condition and the control condition, *B* = −0.24, *p* = .53, odds ratio = 0.79 (see Figure 1). Mediation analysis revealed that the effect of exemplar regret on offer in the fair behavior condition was fully mediated by an increase in anticipated regret and a reduction in anticipated pride (see Figure 2).[Fig-anchor fig1][Fig-anchor fig2]

### Discussion

The results provide support for our theoretical prediction that exemplar emotion influences participants’ allocation behavior by affecting participants’ anticipation of how they would feel. It could be argued that the results of Studies 1A and 1B are the result of strategic inferences about what kind offers would be rejected, rather than anticipated emotions. In that view, the exemplar’s behavior and expressed emotion may have influenced perceived norms of what kind of offers are likely to be rejected, and participants’ anticipated emotions may have merely correlated with the expected outcome of the game. Therefore, in Study 2, we decided to switch from the UG to the dictator game (DG; Kahneman, Knetsch, & Thaler, 1986). The DG does not give the responder the opportunity to reject the allocator’s offer; therefore, there is no strategic component to the allocator’s behavior.

Exemplar emotion did not have an effect on allocation behavior in the unfair condition. Given that only very few participants (1.5%) made offers lower than or equal to 45:5 (see Figure S4 in the supplemental materials), we reasoned that the offer of the exemplar in the unfair condition may have struck participants as surprising and may have thereby overshadowed the influence of exemplar emotion. To test this reasoning, we ran a pilot study (reported as supplemental material S6) and established that exemplar emotion had the predicted effect in the unfair condition when this was varied in the context of less extreme unfair behavior (35:15 instead of 45:5). Therefore, we used the 35:15 division as the example of unfair behavior in Study 2.

We also increased the relevance of the exemplar’s emotion and enhanced the realism of the experimental task. In the context of what appeared to be an online gaming environment, the exemplar was now presented as the participant’s opponent in an initial DG, rather than being a player in another unrelated game (as was the case in Studies 1A and 1B). Exemplar behavior was manipulated in a first DG through the offer that participants received (25:25 or 35:15). Exemplar emotion was manipulated using a filmed message, ostensibly coming from the other player. Afterward, participants were allocators in a second DG, which was independent from the initial DG and played with a different person, and their allocation behavior in that second game served as the main dependent variable.

## Study 2

### Method

#### Participants and design

The study had a 2 (exemplar behavior: fair vs. unfair) × 2 (exemplar emotion: pride vs. regret) between-subject design. Participant recruitment was achieved in the same way as in the previous studies. There were 368 participants who completed the study (*M*_*age*_ = 44.93 years; 55% female).

#### Materials and procedure

The materials and procedure were similar to those of Studies 1A and 1B. After providing demographic information and completing a measure of SVO (Murphy et al., 2011), participants received detailed explanations about the rules of the DG, the roles of the allocator and the receiver, and the fact that they would be paired with two different players in two separate games. They were informed that two randomly selected participants would receive the monetary equivalent of the tokens they won in the game, with each token worth £1. We told participants that the study was concerned with participants’ thoughts and feelings about the game and communication between players. Because of this, filmed “game reviews” would be sent between the players. After comprehension checks, participants entered what appeared to be a virtual online environment. Participants were assigned a pictorial gamer icon (“the bird”) and played their first game against “the fish.”

In the first game all participants were receivers, although the allocation to roles was ostensibly determined by chance. In the fair offer condition, the other player offered the participant 25 of the available 50 tokens. In the unfair offer condition, participants were only offered 15 of the 50 tokens. After the game, participants viewed a short film that contained the exemplar emotion manipulation; here, the fellow player expressed either pride or regret about her/his decision. The gender of participant and exemplar was always the same. After the video, participants completed manipulation checks and indicated (on scales from 1 = *not at all* to 5 = *very much*) how they would feel if they were to divide the tokens in the same way as the allocator in the first game had. These ratings served as measures of anticipated pride (*pleased, proud, satisfied*; α = .93) and anticipated regret (*regretful, sorry*; α = .92).

In the second game, participants acted as allocators (a role that again appeared to be determined by chance) and played against a different player (“the snail”). Participants received 50 tokens and were asked to make an offer to the receiver. The number of tokens they offered was the main dependent variable. After making their offer, participants were asked to provide their own game review in written text (supposedly because of a webcam failure), answered a second manipulation check, and completed a measure of closeness to the person in the video (Aron et al., 1992). Finally, participants were thanked and debriefed.

### Results

#### Participants and data treatment

Twenty-one participants reported problems with playing the video. In postexperimental open-ended comments, another six participants showed clear insight into the study design and hypotheses. These 27 participants were excluded, and 341 participants remained for analysis. A histogram of the distribution of offers is reported in the supplemental materials (S7). We again dichotomized the offers made in the second game. Offers fewer than 25 tokens were recoded as *unfair offers* (33.1%) and offers of ≥25 tokens were recoded as *fair offers* (66.9%). We ran a logistic regression with exemplar behavior (fair vs. unfair), exemplar emotion (regret vs. pride), and gender as predictors. Analyses of the manipulation check data showed that the manipulations were successful. Results are reported in the supplemental materials (S8).

#### Offer level

**Figure 3 fig3:**
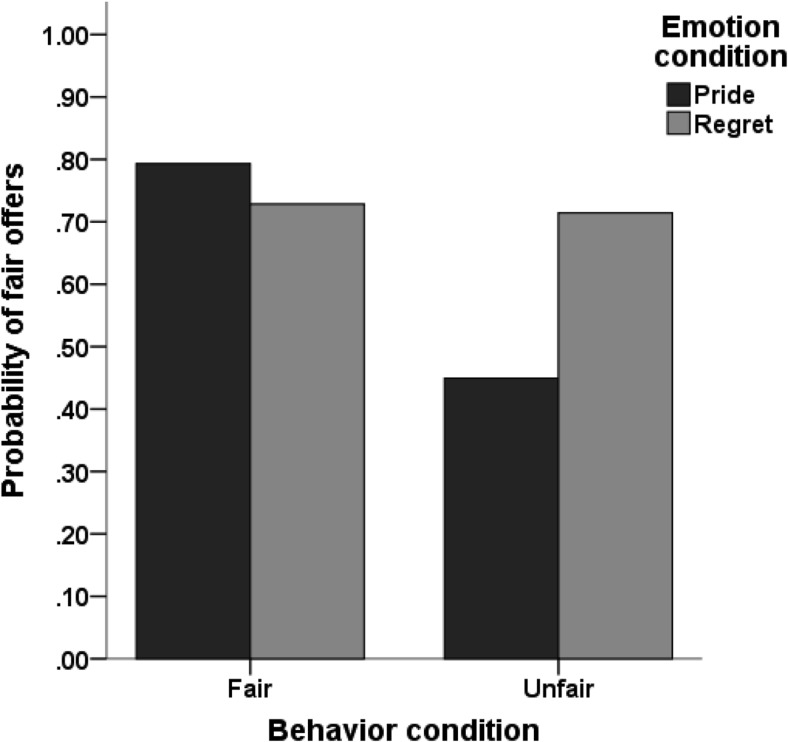
Predicted probabilities of fair offers as a function of exemplar behavior and exemplar emotion (Study 2).

**Figure 4 fig4:**
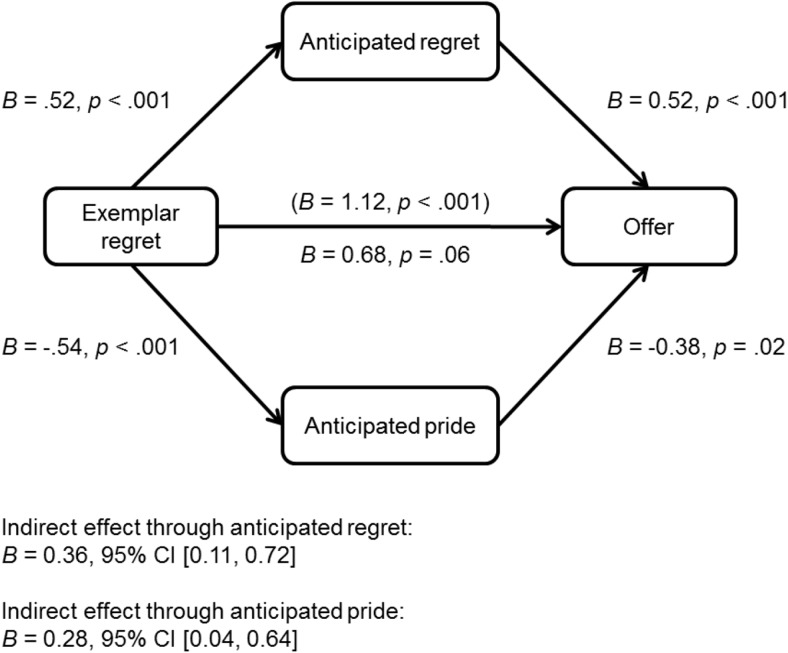
Indirect effect of exemplar regret (compared to pride) on odds of making a fair offer through self-reported anticipated regret and pride in the unfair behavior condition (Study 2).

In line with predictions, there was a significant interaction between exemplar behavior and exemplar emotion, *B* = −1.50, *p* = .002, odds ratio = .22. Follow-up analyses showed that participants who had received an unfair offer were more likely to make a fair offer when the exemplar had expressed regret rather than pride, *B* = 1.12, *p* < .001, odds ratio = 3.05 (see Figure 3). There was no effect of exemplar emotion for participants who had received a fair offer, *B* = −.38, *p* = .30, odds ratio = .68. Mediation analysis further revealed that the effect of exemplar regret on offer in the unfair condition was mediated by an increase in anticipated regret and a reduction in anticipated pride (see Figure 4). In addition, in the fair condition—despite the lack of a direct effect of exemplar emotion on offer—there were significant indirect effects of exemplar emotion (regret compared to pride) on offer through anticipated regret, *B* = −.18, 95% confidence interval [–.52, −.005], and anticipated pride, *B* = −.15, 95% confidence interval [–.46, −.002].[Fig-anchor fig3][Fig-anchor fig4]

### Discussion

Participants who had received an unfair offer in the first game were less likely to make an unfair offer in the second game when the exemplar expressed regret rather than pride. Moreover, the indirect effects of exemplar emotion on offer through anticipated emotions were significant, demonstrating at least partial mediation. Although there was no direct effect of exemplar emotion on offer in the fair condition, the indirect effects via anticipated emotions were significant and in the expected direction. This is consistent with the findings of Studies 1A and 1B.

Extending the findings of Studies 1A and 1B, effects of exemplar emotions were found when participants did not have to make strategic decisions about the level at which offers might be rejected. In Study 2, the emotions of the exemplar did not provide information about the kind of offers that might be accepted. Instead, the expressed emotions only carried information about the kind of feelings that the participant might experience if they were to behave similarly to or differently from the exemplar. Therefore, Study 2 provides further support for an explanation in terms of anticipated emotions.

One structural difference between Study 2 and Studies 1A and 1B was that participants played two consecutive games, first as a receiver and then as an allocator. Being on the receiving end of a fair division of resources might have made a fair division particularly salient and therefore made it more difficult for participants to act unfairly themselves. This could help explain why the influence of exemplar emotion was less pronounced in the fair behavior condition of Study 2.

It could be argued that the different results in the unfair condition between Studies 1A and 1B and Study 2 are due to the differences between the UG and the DG. However, we have evidence that speaks against this. We ran another study that was highly similar to Study 2 except for the fact that participants played the UG rather than the DG. As in Study 2, participants who had received an unfair offer (and had accepted this offer) were less likely to make an unfair offer themselves when the exemplar expressed regret rather than pride (see supplemental material S9).

It might also be thought that the lower percentage of fair offers made in the unfair/pride condition reflects participants’ annoyance at having received an unfair offer in the previous game. The measure of perceived closeness to the exemplar speaks to this possibility. If unfair offers made by participants reflected annoyance, then one would expect unfair offers to be related to low closeness on the grounds that you would not feel close to someone whose actions annoyed you to the extent that you felt like treating another person unfairly. However, in the unfair/pride condition, participants who made an unfair offer felt closer (*M* = 3.31) to the exemplar than did participants who made a fair offer (*M* = 2.27), *F*(1, 87) = 8.14, *p* = .005. This supports an explanation in terms of social appraisal rather than annoyance.

## General Discussion

We found support for the prediction that emotion expressed by a third party about a decision that he or she has taken influences the decision taken by someone who observes that emotional expression. A third-party exemplar who expresses regret about acting fairly elicits less fair behavior on the part of an observer than does an exemplar who expresses pride about acting fairly (Studies 1A and 1B); likewise, an exemplar who expresses regret about acting unfairly elicits more fair behavior than does an exemplar who expresses pride about acting unfairly (Study 2). The studies also shed light on the process underlying these effects. We argue that others’ emotions about their decisions shape the behavior of observers by influencing the emotions that observers anticipate experiencing if they were to act in the same way (Baumeister et al., 2007).

Social psychology has a long tradition of demonstrating the influence of exemplars on behavior (e.g., Asch, 1955; Bandura, 1965; Latané & Darley, 1968; Sherif, 1936), and in developmental psychology it is well established that caregivers’ emotion expressions help to shape how children deal with ambiguous situations (Blair, 2003; Sorce, Emde, Campos, & Klinnert, 1985). Our research suggests that the emotions of exemplars are also important resources for social learning in adult life and that they exert their influence through a combination of social appraisal and anticipated emotions.

In the current research, we tested our predictions concerning social appraisal and anticipated emotions in the context of two economic games. One question that could be asked is whether the predictions would have been supported if we had used other games, such as the trust game (Berg, Dickhaut, & McCabe, 1995), or real economic decisions or decisions involving resource allocation that are not straightforwardly economic in nature (e.g., volunteering time, effort, or expertise on behalf of others). There is no a priori reason to suppose that the process identified in the present research is one that is limited to the economic games we used or indeed to economic games in general. Rather, we propose that social appraisal and anticipated emotions may be critical in the formation and maintenance of social norms in a broad array of behavior domains. The expression of self-conscious and sociomoral emotions demonstrates the values and behaviors that are considered appropriate within a particular social group whereas the anticipation of these emotions on the basis of observing others’ expressions guides behavior in a way that is consistent with these norms. On a broader level, this reciprocal relationship between expression and anticipation of emotions might contribute to the maintenance of social order (Hochschild, 1979; Thoits, 2003).

## Conclusion

Our general argument is that how others are seen to appraise and respond emotionally to their own actions will influence the emotions that observers anticipate experiencing and that these anticipated emotions guide observers’ subsequent behavior. When the Gordon Gekko character in the film *Wall Street* states that “greed . . . is good,” he communicates a message that acting in one’s own interest is something positive, and he reinforces this by implying that it is something in which individuals can take pride. In justifying his own behavior, he thereby helps to establish a social norm that acting in one’s own interest without concern for the welfare of others is appropriate. Conversely, public displays of remorse about self-enrichment (like the quote from Dennis Kozlowski presented in the introduction) may serve an important societal function in that norms about fairness are reinstated after transgressions of these norms. We should not be surprised to find that expressions of emotions in situations such as these have an influence on the behavior of witnesses.

## Supplementary Material

10.1037/xge0000036.supp
