# Supplementary material for: The Social Power of Regret: The Effect of Social Appraisal and Anticipated Emotions on Fair and Unfair Allocations in Resource Dilemmas
Source: J Exp Psychol Gen. 2014 Nov 10;144(1):151–7. doi: 10.1037/xge0000036 (PMC4312135; doi:10.1037/xge0000036)
Supplement: Supplementary file 2 [file XGE-2014-0210_supplemental-material.reformatted.v2.docx]

**Supplemental material**

**S1. Pilot Study 1**

We ran a pilot study (*N* = 113, after 4 exclusions) to establish whether providing participants with information about the decision made by an exemplar and the emotion felt about this decision would affect participants’ own allocation decisions in the UG. Participants read a ‘thought protocol’ ostensibly provided by a previous participant who made either a 50-50 split (fair behavior condition) or a 90-10 split (unfair behavior condition), and – orthogonal to this behavior manipulation – felt either pride or regret about this decision (emotions were not mentioned in the control condition). Details of these manipulations are provided in the main text when describing the methods of Study 1A and Study 1B and below in S2.

When analyzing the number of fair decisions made by participants, we found there was a significant interaction between exemplar behavior and exemplar emotion, *p* = .038. In the fair behavior condition there was a marginally significant effect of exemplar emotion, *p* = .073, but there was no effect of exemplar emotion in the unfair behavior condition. In line with predictions, exemplar regret decreased the odds of a fair offer in the fair behavior condition compared to exemplar pride, *B* = -1.98, *p* = .022; odds ratio = 0.14. This pilot study established that providing participants with information about a previous allocator’s decision and emotions about this decision influenced participants’ own decisions when they came to play the UG as allocators.

**S2. Study 1A – Wording of manipulations**

*Fair/pride condition*: “I feel good about how I played the game. I feel proud that I chose to be fair despite the fact that this meant not making some additional profit. I feel pleased with my decision.”

*Fair/regret condition*: “I feel bad about how I played the game. I feel sorry that I chose to be fair and did not make some additional profit. I regret my decision.”

*Unfair/pride condition*: “I feel good about how I played the game. I am proud that I chose to make some additional profit despite the fact that this meant not being fair. I feel pleased with my decision.”

*Unfair/regret condition*: “I feel bad about how I played the game. I feel sorry that I chose to make some additional profit and was not fair. I regret my decision.”

**S3. Study 1B – Example video recordings**

Two drama student actors (1 male and 1 female) played the exemplars. Their expressions of pride or regret were accompanied by specific facial movements and postures that further supported their verbal statements: In the pride condition the exemplars smiled and tilted their head upwards; in the regret condition the exemplars looked down and adopted a slumped posture. Examples are provided as .mpg files:

MA_25-25_pride.mpg (male exemplar expressing pride having been fair)

FA_25-25_regret.mpg (female exemplar expressing regret having been fair)

FA_45-5_pride.mpg (female exemplar expressing pride having been unfair)

MA_45-5_regret.mpg (male exemplar expressing regret having been unfair)

**S4. Studies 1 A and 1B – Distribution of offers**


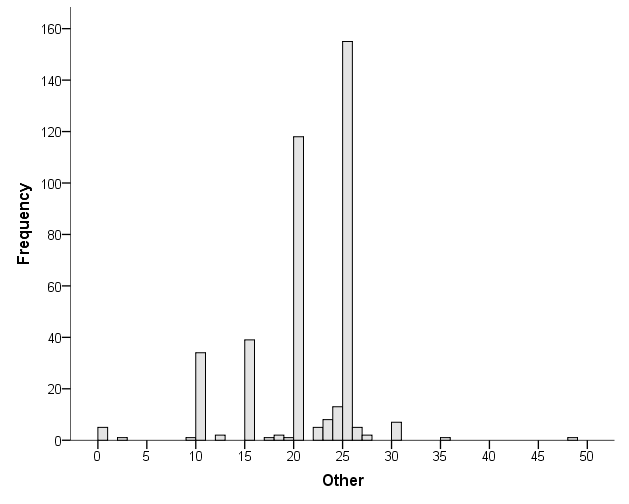
*Figure S4*. Distribution of MUs offered to other player (data pooled from Studies 1A and 1B).

**S5. Studies 1A and 1B – Manipulation checks**

As manipulation checks participants were asked to report the offer that the allocator made in the transcript, and the extent to which this individual 1) regretted his/her decision and 2) was proud of his/her decision on a 5-point scale where 1 = *not at all* and 5 = *extremely*. In total 82.8% of participants reported the correct number of MUs shared by the exemplar in the thought protocol. For perceived pride, a 2(Gender) by 2(Behavior: fair, unfair) by 3(Emotion: pride, regret, control) ANCOVA, with Study as a covariate, revealed the expected main effect of exemplar emotion, *F*(2, 384) = 157.03, *p* < .001, η^2^_p_ = .45. Perceived pride was significantly higher in the pride condition (*M* = 4.46, *SD* = 0.94) than in the regret (*M* = 2.17, *SD* = 1.22) and control conditions (*M* = 3.80, *SD* = 1.00), both *p*s < .001. Although the two-way interaction between exemplar behavior and exemplar emotion was significant, *F*(2, 384) = 3.45, *p* = .033, η^2^_p_ = .02, pairwise comparisons with Bonferroni correction revealed that in both behavior conditions participants accurately identified more pride in the pride condition. No other effects were found. For perceived regret, too, a 2 x 2 x 3 ANCOVA, with Study as a covariate revealed the expected main effect of exemplar emotion, *F*(2, 381) = 303.04, *p* < .001, η^2^_p_ = .61. Participants identified more regret in the regret condition (*M* = 4.19, *SD* = 1.06) than in the pride (*M* = 1.41, *SD* = 0.93) or control conditions (*M* = 1.69, *SD* = 1.00), both *p*s < .001. Neither the main effect of exemplar behavior nor the interaction between exemplar behavior and emotion were significant. We concluded that the manipulations were successful.

**S6. Pilot Study 2**

Given that only very few participants (1.5%) made offers lower or equal to 45:5, we reasoned that the offer of the exemplar in the unfair condition may have struck participants as surprising and have thereby overshadowed the influence of exemplar emotion. To test this reasoning we ran a pilot study (*N* = 158, after 24 exclusions) to establish whether participants would be influenced by exemplar emotion when this was varied in the context of less extreme unfair behavior (35:15 instead of 45:5). All participants were exposed to an exemplar who reported having allocated 50 MUs in the proportion 35:15 between self and other. In the pride conditions the exemplar expressed pride about the allocation; in the regret conditions the exemplar expressed regret about the allocation. We also tested whether framing the allocation as fair or unfair (by having the exemplar describe the allocation as ‘fair’ or ‘unfair’) would moderate the impact of exemplar emotion. In the control condition no emotions were mentioned, and the allocation was not framed as fair or unfair. We predicted that exemplar regret about a 35:15 allocation framed as unfair would lead to more fair offers and exemplar pride about a 35:15 allocation framed as unfair would lead to fewer fair offers. The materials and procedure were identical those used in Study 1B except for the offer and how this was framed in terms of fairness.

Exemplar regret increased the odds of a fair offer compared to exemplar pride, *B* = 1.10, *p* = .008, odds ratio = 3.01. This effect was not moderated by framing of the allocation, *B* = -0.74, *p* = .38, odds ratio = .48. However, in line with our predictions the effect of exemplar regret was stronger in the condition where the 35:15 offer was framed as unfair, *B* = 1.39, *p* = .021, odds ratio = 4.01, compared to when the offer was framed as fair, *B* = 0.65, *p* = .27, odds ratio = 1.92.

Exemplar regret about unfair behavior therefore had the expected effect on allocation behavior when a less extreme example of unfair behavior was used (35:15 rather than 45:5). This suggests that the lack of effects in the unfair behavior condition across Studies 1A and 1B was due to the unfair behavior being too extreme. We therefore used the 35:15 division as the example of unfair behavior in Studies 2 (main text) and 3 (see S9 below).

**S7. Study 2 – Distribution of offers**

*
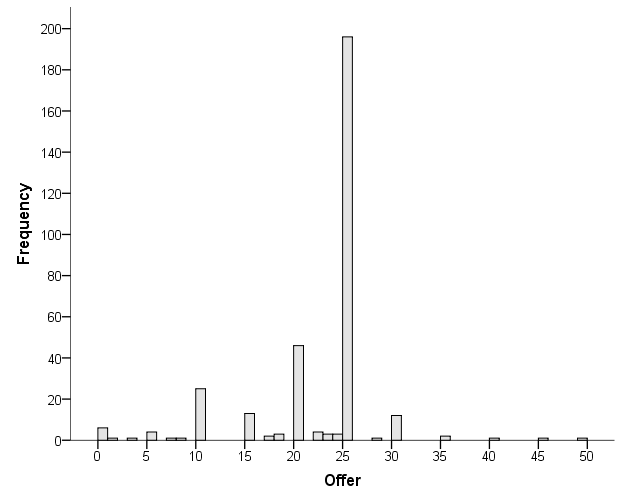
Figure S7*. Distribution of MUs offered to other player (Study 2).

**S8. Study 2 – Manipulation checks**

Immediately after seeing the video, participants were asked to report how the tokens had been allocated and the extent to which they thought the fellow player 1) regretted and 2) was proud of his or her decision (on scales from 1 = *not at all* to 5 = *very much*), as manipulation checks. In total 92.4% of participants reported the correct number of MUs shared by the exemplar in the video. Participants reported having perceived more pride in the pride (*M* = 4.47, *SD* = .95) than in the regret (*M* = 1.97, *SD* = 1.08) condition, *F*(1, 333) = 521.42, *p* < .001, η²_p_ = .61, and more regret in the regret (*M* = 4.18, *SD* = .97) than in the pride (*M* = 1.49, *SD* = 1.02) condition, *F*(1, 333) = 616.21, *p* < .001, η²_p_ = .65. For perceived pride, there was also a main effect of offer: Participants in the fair condition perceived more pride (*M* = 3.39, *SD* = 1.57) than did participants in the unfair condition (*M* = 3.05, *SD* = 1.63), *F*(1, 333) = 9.42, *p* = .002, η²_p_ = .03. No other main effects or interactions were significant. We concluded that the manipulation had been successful.

**S9. Study 3**

We ran a third study (*N* = 266, after 62 exclusions based on the same criteria as in Study 2) that was highly similar to Study 2, except that participants played the UG rather than the DG and that gender of participant and exemplar in the video was fully counterbalanced (gender of the exemplar in the video did not have any main or interaction effects, all *p*s > .10). Participants again played the game twice, with two different players. In the first game they took the role of receiver, which was ostensibly determined by chance. In the fair condition, participants were offered 25 of the available 50 tokens; in the unfair condition, participants were offered 15 of the 50 tokens. Unlike in Study 2, participants could either accept or reject the offer. They then viewed two filmed messages that were ostensibly recorded *before* acceptance/rejection of the offer was communicated. In the first filmed message the fellow player reiterated the rules of the UG and the offer he or she had made. A second film contained the exemplar emotion manipulation; the fellow player expressed either pride or regret about her/his decision. In the second game, participants acted as allocator (a role again ostensibly determined by chance) and played with another opponent. The number of tokens participants offered was the main dependent variable. The manipulation checks, the measures of anticipated pride (α = .92) and regret (α = .90), and other measures were identical to those used in Study 2.

Examination of the data suggested that the impact of exemplar behavior differed depending on whether participants decided to accept or reject the offer in the first game. Perhaps unsurprisingly, only 2 participants rejected the offer in the fair condition, whereas 71 out of 130 participants did so in the unfair condition. In our analyses we therefore made a further distinction between those who accepted and those who rejected the unfair offer.

Analyses showed that participants who had accepted an unfair offer were more likely to make a fair offer when the exemplar had expressed regret rather than pride, *B* = 1.24, *p* = .04, odds ratio = 3.45. There were no effects of exemplar emotion for participants who had rejected an unfair offer, *B* = -0.58, *p* = .28, odds ratio = .56, or for participants who had received a fair offer, *B* = -0.19, *p* = .64, odds ratio = .83.

As in Study 2, these findings show that exemplars’ regret about making an unfair offer reinforced fair behavior. The fact that the expressed emotion did not have an effect on participants who rejected the unfair offer further suggests that the act of rejecting such an offer—presumably on the grounds that it was seen as unfair—has the effect of reinforcing the fairness norm, thereby making it more difficult for participants to then act unfairly themselves. Similarly, having received a fair division of resources in the first game may have made a fair division particularly salient when participants acted as allocators in the subsequent UG. This may have limited the scope for any additional influence of exemplar emotion in the fair behavior condition.
